# Co-creation Processes and Urban Digital Twins in Sustainable and Smart Urban District Development - Case Kera District in Espoo, Finland

**DOI:** 10.12688/openreseurope.17791.1

**Published:** 2024-06-28

**Authors:** Jani Tartia, Mervi Hämäläinen

**Affiliations:** 1 Centre of excellence for sustainable development, City of Espoo, Espoo, 02270, Finland; 2 Digital service development and knowledge management, City of Espoo, Espoo, 02270, Finland

**Keywords:** Urban development, co-creation, brownfield development, positive energy districts, digital twins

## Abstract

In the context of climate change mitigation, calls for methods that can facilitate co-creative practices and processes between different stakeholders in the development of sustainable and climate-neutral urban districts have been increasingly expressed in recent years. This has included calls for collaborative, accessible, transparent and open tools that can facilitate urban development processes and engage different stakeholders in the different phases and stages of an urban district development process towards shared targets on sustainability and emission reduction. In this paper, we present and examine two practical tools, 1) a co-creation model for developing positive energy district (PED) solutions, and 2) a digital twin tool for shared data sharing and collaboration, which were developed and utilized recently in two development projects focusing on Kera district, Espoo, Finland. The Kera district is a current brownfield area, which, since the early 2020s, has been undergoing a transformation from a former industrial and mass logistics area into a future mixed-use urban district with significant emphasis on sustainability and circular economy targets. We utilize actor-network theory (ANT) to explore the human and the non-human actors and their interrelations related to the district’s development. Additionally, we seek to understand the networks that emerge within both the co-creation process and the virtual urban digital twin environment and the role these tools have in supporting the formation and facilitation of multi-stakeholder co-creation networks. Finally, we aim to examine the advantages and disadvantages of integrating a regional urban digital twin and the urban co-creation process to enhance sustainability in urban development projects.

## Introduction

The complexity of the challenges related to climate change adaptation in cities – for example in the energy, circular economy and sustainable mobility sectors – have highlighted the need for participatory processes in urban development that engage different (local) stakeholders in the different phases of an urban area or district’s development process towards sustainability and climate-neutrality. In the European context calls for participatory approaches in urban development are increasingly expressed by many EU development programmes, such as Built4People, Horizon 2020 Europe, and the New European Bauhaus initiative. ‘Co-creation’ – which, as a term, has roots in business and marketing research – has especially been a recent ‘buzzword’ through which urban development has been approached, referring to a more-than-collaboration type of processes of working together towards a shared goals through innovative solutions, in this case, on the complex issues on sustainability and climate-neutrality ( Davis & Andrew, 2017; Leino & Puumala, 2021; Lund, 2018). Different co-creation approaches, in general, underline network-like typology, openness, transparency and diverse engagement toolkits and methods, which are among some of the key differences in relation to more common participatory processes, which might be seen as inefficient, exclusive and less-transparent (see Leino & Puumala, 2021; Torfing & Ansell, 2021).

In urban development, the topic of co-creation is not a new one – the principles of participation go back at least to the 1970s (see Arnstein, 1969; Bäcklund & Mäntysalo, 2009; Gaber, 2019; Healey, 1993; Lund, 2018; Lynch, 1984; Ruiz-Mallén, 2020) – but the calls for more holistic participatory processes and the empowerment of different stakeholders and social groups have become louder in recent decades, especially in relation to topics such as urban renewal projects ( Davis & Andrew, 2017), smart city development ( Leino & Puumala, 2021), and sustainable urban development after the United Nations’ Rio Declaration in 1992 ( UN, 1992) (see Ahvenniemi *et al.,* 2017). Carbon-neutrality, circular and sharing economy principles, inclusivity and accessibility, and just transition, are some of the key system level ‘wicked problems’ (see Skaburskis, 2008) in sustainable urban development, and co-creation has emerged as a potential tool to provide solutions for these issues ( Davis & Andrew, 2017; Ruiz-Mallén, 2020). Through a wider engagement of both organizations and individual citizens, more inputs can be gathered to guide the development processes, rise different perspectives, concerns and possibilities for discussion, and to produce legitimacy and popular acceptability for the actions through engagement ( Røiseland, 2022; see also Bäcklund *et al.,* 2017). At the same time, however, co-creation as a mere statement or a phrase does not automatically guarantee an actually inclusive or open process ( Davis & Andrew, 2017; Leino & Puumala, 2021), and the engagement does not necessarily reach all facets of the society equally ( Røiseland, 2022).

In this paper, we approach participative co-creation processes for sustainable urban development through the lens of networks. Our objective is firstly to examine the types of processes and tools that could facilitate the formation of robust actor networks and support collaboration of different stakeholders in co-creation processes within the context of redeveloping brownfield areas. Drawing on the *actor-network theory* (ANT), urban development processes can be understood as complex heterogeneous networks, where different human and non-human, or social and material, *actants* operate in reciprocal relations with varying level of intensity and connectedness ( Rydin, 2012; Rydin & Tate, 2016). To achieve the study objective, we conducted a combined examination of two specific tools:1) a co-creation model designed to facilitate a co-creative urban development process on a district level, and 2) a digital twin tool for collaboration and data sharing. These tools were developed and experimented in two separate EU-funded development projects within our case study area, the Kera district in Espoo, Finland. Additionally, we seek to understand the networks that emerge within both the co-creation process and the virtual urban digital twin environment and the role these tools have in supporting the formation and facilitation of multi-stakeholder co-creation networks. Finally, we aim to examine the advantages and disadvantages of integrating a regional urban digital twin and the urban co-creation process to the enhance sustainability in urban development projects.

This paper sets its empirical foundation on two EU funded development projects on urban transformation, co-creation, green transition and digitalization. The first project (titled *SPARCS Sustainable energy Positive and zero cARbon CommunitieS*, 2019–2024, funded by the EU Horizon 2020 programme) explored different co-creation processes for developing sustainable and resilient *positive energy districts* (or PEDs), which requires the involvement of a diverse set of different types of (local) actors in various roles and in various phases of the district’s planned life cycle. The second project (titled *Implementation pathway for environments that accelerate sustainable growth*, 2021–2023, funded by the REACT-EU) investigated and compiled information of digital environments that facilitate communication and stakeholder collaboration on virtual platforms. The paper presents the main insights gained from these two projects on the co-creation processes, and their relation to an urban brownfield redevelopment case of the Kera district, which has acted as case example in both projects. The paper is complemented with insights gained from an additional stakeholder workshop, which was organized to combine the co-creation process and urban digital twin developed during these two projects to explore the potential of co-creation processes utilizing virtual tools for collaboration and communication.

The structure of the paper is as follows. First, we present the theoretical background, the actor-network theory, which serves as the foundation for observing the participants engaged in participatory and co-creative processes within the urban environment. Thereafter, we explore the linkages between ANT and urban development co-creation processes, followed by an overview of the case study context, the Kera district. Then, we present the co-creation processes from the two aforementioned projects. The main insights are then discussed, and the relations between the introduced tools and ANT are presented. Final thoughts and prospective new research topics are presented in the end.

## Actor network theory and co-creation of urban districts

The actor network theory has its origin in science and technology studies. Its essence lies in *human* (individuals) and *non-human* (e.g. machines, texts, computers) entities and how these entities assemble and associate, expand and decline over time. ( Fenwick & Edwards, 2012.) One of the notable benefits of ANT is that it allows researchers to observe complex socio-technical systems through the heterogenous *human and non-human* entities that operate and influence in society ( Latour, 1996). Law (1992) even highlights that societies and organizations cannot exist without the interplay of these human and non-human actors.

Within the framework of ANT, the human and non-human entities are called as *actants* or *actors* and they are seen as a source of action. Latour (2005: 71) indicates that ‘any thing that does modify a state of affairs by making a difference is an actor’. The actors are mentioned as mediators that *do* things and which can form free associations with other actors. Through interaction and communication, actors form connections and build heterogeneous networks. Latour (1996) further suggests that actor network surrounds itself with explanatory resources. The explanatory resources demonstrate how one element or actor holds and connects with each other in the actor network. Explanatory resources form the ground for the growth and expansion of the actor network and give locale for understanding the relationships and interactions between actors.

From a critical point of view, the ANT approaches have been criticized for not taking certain social (such as race, gender and ethnicity), historical and cultural aspects into proper consideration when examining the network, its actants and their interrelations ( Müller, 2015). Further, ANT is criticized of its inability to think the specificity of the event intertwined with performance and practise ( Smith & Doel, 2011).

In urban studies ANT has had a significant influence on exploring the (socio-technical) networks and actors present in the modern urban environment. ANT has been deployed to deepen the understanding of complex actor networks whether they involve economic innovations, social relationships, or environmental interdependences within the city. ( De Munck, 2017.) The deployment of ANT in urban studies has been justified because it not only emphasises the presence of human actors in networks, but also considers inanimate objects and entities, such as technological tools and solutions, as equal participants in the complex urban context ( De Munck, 2017; Farías & Bender, 2009). Further, ANT is seen to assist urban researchers to identify the relationships of the objects and explore the associations and interconnections between these human and non-human objects ( Farías & Bender, 2009), the different power dynamics inside the networks ( Rydin & Tate, 2016), and how the ‘relationships between actants are forged, negotiated and maintained.’ ( Rydin, 2012: 25). The networks, from an ANT perspective, are always mobile, meaning that the connections between the actors are dynamically changing and transforming constantly – alternatives for the ‘network’ term are *assemblage* and *rhizome*, which aim to capture the temporal and dynamic nature of the networks ( Rydin & Tate, 2016). ANT emphasizes the continuously emergent nature of the networks, and that the network, as a whole, is more than the sum of its parts ( Müller & Schurr, 2016).

In this study ANT is deployed to observe those human and non-human actors that emerge in our case study area, the Kera district. As ANT assists the researchers in depicting the actors and observing the translations between them throughout different stages of the urban co-creation process ( Cvetinovic *et al.,* 2017), our aim is to identify those human and non-human actors and explore their interrelations within the context of both the physical and the virtual Kera district. Färber writes, while emphasising the temporal, virtual and continuously-in-the-making nature of urban assemblages, that ‘working with ANT shows how agency is distributed within the socio-material situations of creating a city and highlights the contingency and multiplicity of these socio-material situations’ ( 2020: 264). Further, the objective is to deepen our understanding of the role played by non-human elements in facilitating the co-creative urban development process together with co-creative methods and digital twin technologies.

## Co-creation of urban districts through the lenses of ANT

Participatory approaches to urban development have been around at least since the 1970s. The aim of participation is to utilize the information and identified needs and wants of individuals, groups and organizations. (See e.g. Arnstein, 1969; Bäcklund & Mäntysalo, 2009; Gaber, 2019; Leino & Puumala, 2021; Lund, 2018; Ruiz-Mallén, 2020.) There is a long frictional history between urban development and local resident participation, also grassroot-level activism and resistance, as the needs of the people have often been different than the ‘grand designs’ presented by the planners (see Jacobs, 1961). What exactly is meant by *participation* in urban development processes has evolved during the decades, and the role of the participants has changed from a passive information provider towards more of an active collaborator and local expert ( Bäcklund & Mäntysalo, 2009; Leino & Puumala, 2021; Ruiz-Mallén, 2020). As cities are made of heterogeneous individuals and social groups, the needs, wants and desires related to the built environment are different and varied, which mean that there is always competing interests present in urban development, and thus, as the planning process can never have the ‘full’ picture or data but it is always incomplete ( Bäcklund & Mäntysalo, 2009; Bäcklund *et al.,* 2017).

Co-creation has emerged in recent years as an approach, which has been seen as a way to alleviate some of these potentially frictional issues through working directly with the different groups towards defining shared goals and working processes. Co-creation is rooted in business and marketing research, where it has been originally used to design products together with the customers ( Davis & Andrew, 2017). The term ‘co-production’ is also used almost interchangeably with co-creation ( Ruiz-Mallén, 2020). In the urban context, co-creation, in general, has come to mean especially different collaborative ways to design the built environment with the local residents and other stakeholders. The citizen-centric co-creation process, for example, are often manifest as different types of *urban living labs*, which, as local hotspots, are used to identify and solve local and contextual issues ( Leino & Puumala, 2021; Lund, 2018; Puerari *et al.*, 2018). Co-creation is also used with local organization and business ecosystems to jointly draft strategic goals or to conduct shared operations, and to developed ‘partnership’ type of relations ( Ruiz-Mallén, 2020). Co-creation processes aim for inclusivity, openness and transparency but the research on the topic indicates that we still have a long way to go towards inclusive co-creation that supports the participation, engagement and empowerment of different stakeholders equally ( Elkjær & Horst, 2023; Leino & Puumala, 2021; Lund, 2018; Ruiz-Mallén, 2020; Torfing & Ansell, 2021). Co-creation, thus, still generally remains as an under-developed practice ( Leino & Puumala, 2021; also Ruiz-Mallén, 2020). In the next sections, we aim to contribute to this emergent topic through examining closer a brownfield redevelopment case, and what kind of roles different co-creation tools and technologies can have in forming and sustaining the actor-networks related to local urban renewal processes.

The term ‘brownfield development’ is not fixed as it has different interpretations in different countries, and it has evolved through time. In general, it refers to vacant or underutilized industrial or commercial areas that are redeveloped for residential or mixed use. An opposite for brownfield development is ‘greenfield development’, which refers to the development of a previously unbuilt area. ( Adams *et al.,* 2010.) The brownfield area development projects have been born out of the general need for sustainable land use practices, which is done by rearranging and reassigning land uses in growing cities ( Cappai *et al.,* 2019). Industries and modes of production are constantly changing, which renders some industrial areas and their uses obsolete. The vacant or even derelict sites are increasingly seen as an urban planning opportunity rather than as a problem ( Adams *et al.,* 2010). The use of the brownfield areas can help to prevent urban sprawl (as opposite to greenfield development) and to sparse together the urban structure that has previously been separated by industrial zones that are out of limits for most of the populations. However, there are concerns related, for example, to the gentrification of such areas, as the redevelopment can push the previous users of the area to other areas or they can be out of reach of low-income groups (e.g. due to high land prices and high rental prices of apartments/business premises) ( Bryson, 2012).

In recent years, there have been multiple brownfield development cases in different cities in Finland, in which a former predominantly industrial area is redeveloped into a case example of sustainable and smart city development in the context of the local city. These include Kera, an old logistics centre in Espoo (examined in the following sections), Hiedanranta, a former pulp mill factory area in Tampere, and Jätkäsaari and Kalasatama, both former harbour areas in Helsinki. International examples are also aplenty, such as King’s Cross in London, UK, Hammarby Sjöstad in Stockholm, Sweden, and Sluppen in Trondheim, Norway. In many regards, the redevelopment of this types of areas are tightly connected with different smart city, circular economy and sustainable district strategies and visions (see Nylén *et al.*, 2021).

In the next sections we turn our focus to the case example of Kera brownfield district development in Espoo, Finland, and present and discuss two practical co-creation process development cases on a model for co-creation process facilitation and digital twins, which utilized Kera as a pilot area.

## Redeveloping urban brownfield areas – Case Kera

**Figure 1.**
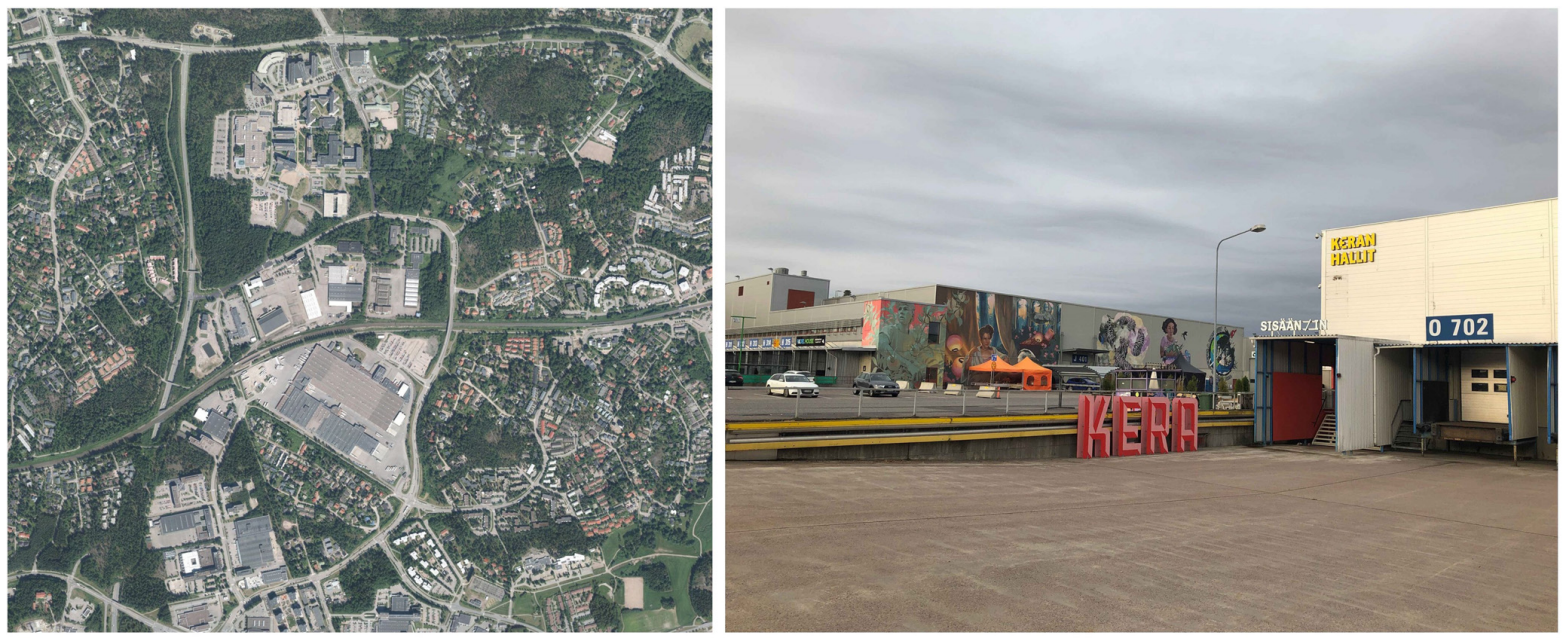
The Kera area. Sources: City of Espoo (left), authors (right).

Kera, located in the middle of the Espoo municipality (approx. 300.000 residents), Finland, is an old brownfield area of 58 hectares, which is currently redeveloped into a new urban district ( Figure 1). The aim of the City of Espoo is to develop Kera into a new sustainable and smart city district for living and working that broadly utilizes carbon-free and circular economy solutions to comply with the city’s carbon neutral 2030 target. The current plans for the area indicate that it will grow into a district for at least 14.000 residents and 10.000 workplaces between the 2020s and 2040s ( City of Espoo, 2024a). The land in the area is mainly privately owned and the city collaborates with the current landowners, construction companies, different service providers, local businesses, and (future) residents in the development of the area towards the defined goals for sustainability and *smart city* principles of data utilization – which have been set as part of different co-creation processes in different stages of the redevelopment planning, facilitated by the city. The construction work for the new Kera began in December 2023 with city-led street construction work, and the redevelopment of the area is expected to last to 2040 and beyond ( City of Espoo, 2024b).

One of the key results that have come out of the collaboration between the different stakeholders in Kera in the planning phase of the district is the ‘Kera area development commitment’ document, which is an attachment to the land use agreement. The commitment, based on a shared vision of the district’s future, describes the general outlines for developing energy, mobility, construction, circular economy and housing solutions that are in line with the city’s carbon neutrality targets and the aims to develop the area into a new kind of exemplary area of sustainable development and circular economy ( City of Espoo, 2024b; see also Nylén *et al.,* 2021). The commitment is the result of numerous development projects of the City of Espoo and other partners, which have developed the shared visions through co-creation workshops and which have also piloted new solutions in the area.

The main development work in the area, in the interim between the ‘old’ industrial district Kera and the ‘new’ sustainable district Kera, is focused on the site of a large logistics centre that operated in the area between the 1960s and 2019 ( Figure 1). The old logistics centre has become the staple of the area and its renewal process, mainly through the temporary and *pop-up* utilization of the vacant spaces under the Keran Hallit (Kera Halls) concept, which is a collaborative effort of the city organization, the third sector (organized as Kera Collective) and the current landowner. The massive logistics halls of the centre, with an indoor floor area of 0,92 ha, have facilitated cultural events and temporary rental of work spaces for local start-ups, small breweries, sports facilities (padel courts, gym, skateboard park), and others. The main logistics hall is to be demolished in 2025 to give space for the new buildings, following the master plan for the area approved by the city council in 2021.

This ‘temporary’ Kera – set between the ‘old’ and the ‘new’ Kera – can, through the lenses of ANT, be seen as a complex network of diverse actants. The logistics halls and their temporary uses form major non-human and material actants, which are combined with diverse human actants, including the hall operators and individuals and groups from the fields of culture, start-up communities, and service operators. The ‘temporary’ Kera network(s) would be an interesting research point on its own. However, in this paper, we focus on the networks related to the redevelopment planning and design of the area, which focus on the shaping of the ‘new’ Kera on the planning table. Kera’s active development through collaboration between the city and current landowners and organizations has acted as a basis for the development of new co-creation tools in two EU funded development projects, presented in the next sections.

## Co-creation process for smart and sustainable urban areas – Case Kera

Sustainability of an urban district can be approached from many angles. The Horizon 2020 funded SPARCS project aimed to develop new solutions for *positive energy districts* (PEDs). As an emergent and evolving concept, there is no single definition for a PED but, in general, it refers to an urban mixed-used district that produces more (renewable) energy than consumes it by making use of smart energy and mobility solutions that are integrated to the built environment, such as renewable energy production and storage, utilization of waste heat, energy efficiency, and electric mobility ( Casamassima *et al.,* 2022; Cheng *et al.,* 2021; Derkenbaeva *et al.,* 2022). The PED concept generally can be seen as one possible approach to reach the different city-level, national and EU-level carbon neutrality targets towards climate-neutral and climate-positive cities (as defined as part of the EU’s Strategic Energy Transition Plan or SET-Plan) through *district level* solutions ( Cheng *et al.,* 2021). As the focus in the PED concept is set on the district level rather than a building-level, this means that active collaboration between different local stakeholders in the area – including citizens, companies, organizations, landowners, service providers and city departments – is required to achieve shared, district-level outcomes as the uses, functions and operations of a district have to be optimized and interconnected to achieve energy savings and energy efficiency (Ibid.; Derkenbaeva *et al.,* 2022). The collaboration between stakeholders is not only relevant in the planning or construction phases of a district’s lifecycle but also in the production and management phases as the different solutions and flows are interconnected ( Cheng *et al.,* 2021).

Whereas there already exists many technological solutions for PEDs, the implications of PEDs for urban planning and design processes and practices have been less examined so far. For these reasons, one tangible aim of the SPARCS project was to develop a ‘co-creation model’ for PED development in the context of Espoo and the Kera brownfield redevelopment district. The main question for developing the model was related to how PEDs can be co-planned, co-produced and co-managed together with the different stakeholders related to the development, investment, construction, maintenance and use of the PED solutions. A co-creation process with the identified stakeholders of the Kera district redevelopment was selected as the method of approach to generate the model. The process to develop the model, or a practical toolbox, aimed to provide information, methods and a general process-description for a structured co-creation process between different stakeholders in developing PEDs and related energy and mobility solutions, which could be utilized in any urban area in any city, not only in the Kera district.

The model development was done through an eleven-month (11) co-creation process, led by the city and a subcontracted consultancy company. The open process comprised of a mixture of case reviews of similar co-creation urban development processes (of which some are mentioned in the text above), benchmarking of methods and tools for co-creation, expert interviews on the topic of sustainable cities and co-creation, multiple Design Sprint workshops, questionnaires and interactive webinars, which were utilized to develop the model together in a close collaboration with the different stakeholders. These stakeholders included local companies, organizations, city department, research institutes and universities, other cities and local citizens. Those stakeholders, who had been part of the Kera development processes earlier, were invited to join the process through direct communication efforts, but the participation was kept open for all interested parties, as presented through open calls on different platforms and sites, including the city’s website. The development process of the model was explorative in style, as it aimed to tackle issues which had not yet been rooted into the day-to-day urban planning and development practices. The model work was finalized in early 2023.

The co-creation process – the identified roles, responsibilities, methods, timeframes and other variables – are depicted through six basic steps of the co-creation process: (1) the identification of the development need and the stakeholders, (2) the mapping of the current situation and the development needs, (3) the setting of objectives, (4) the identification of solutions, (5) project planning, and (6) implementation and deployment. The steps can be utilized, for example, when co-creating PEDs, new sustainable energy systems or shared mobility service ecosystem in a specific area. The different typologies of the areas – geographical, social, environmental, existing infrastructure etc. – create the specific context for the process. These steps depict an ‘ideal’ situation for the process, which in real-life are heavily affected and influenced by the context, the area’s development history (including the existing path-dependencies) and roles of different stakeholders, as place-specific processes do not begin from an empty board but carry the local spatial, social and cultural history and existing narratives.

The process is dependent of a facilitator – such as the city organization – to initiate the process and facilitate the different steps together with the stakeholders. The start of the process can be motivated by other actors as well but the structured process requires the utilization of coordinating resources, which might be unattainable in many development cases. The usefulness of the process is also dependent of the commitment of different stakeholders to the development process. Ideally, the steps of the process can be used to foster the commitment of the relevant stakeholders (short-term, long-term) but it is clear that there are many drivers and elements that have an effect on how the commitment is built up and what kind of rationalities they are based on, whether economic, social or ecological principles, on individual or shared targets, or on short-term or long-term expected gains and benefits.

## Kera digital twin

As part of the *Implementation pathway for environments that accelerate sustainable growth* project *,* one of the objectives was to identify digital environments and applications applicable in the contexts of construction, autonomous mobility, intelligent energy solutions and circular economy. The purpose was to find a digital platform(s), which could serve as virtual environments for communication and collaboration among the stakeholder operating in the Kera district. Additionally, the goal was to find a platform capable of supporting the creation of digital twin for the evolving Kera district.

Digital twin technologies in urban planning and development have gained interest among urban developers. Multiple cities globally produce three dimensional (3D) model of their cities, which forms a foundation for visual urban digital twin. However, 3D city model alone is not enough to provide added value, rather the model needs to be enriched with datasets, both historical and real-time, to augment the potential benefits the virtual digital twins may offer for actors in urban development projects. ( Hämäläinen, 2021.) In the Kera case, the Kera digital twin would set its foundation on the 3D city model, which the City of Espoo produces from its urban districts. Moreover, the aim here was that the Kera digital twin could be enriched with data facilitating the evaluation and visualization of sustainable urban solutions in Kera. The ultimate goal was to have a solution, capable of calculating CO2 emissions and identifying carbon sinks in the Kera district.

**Figure 2.**
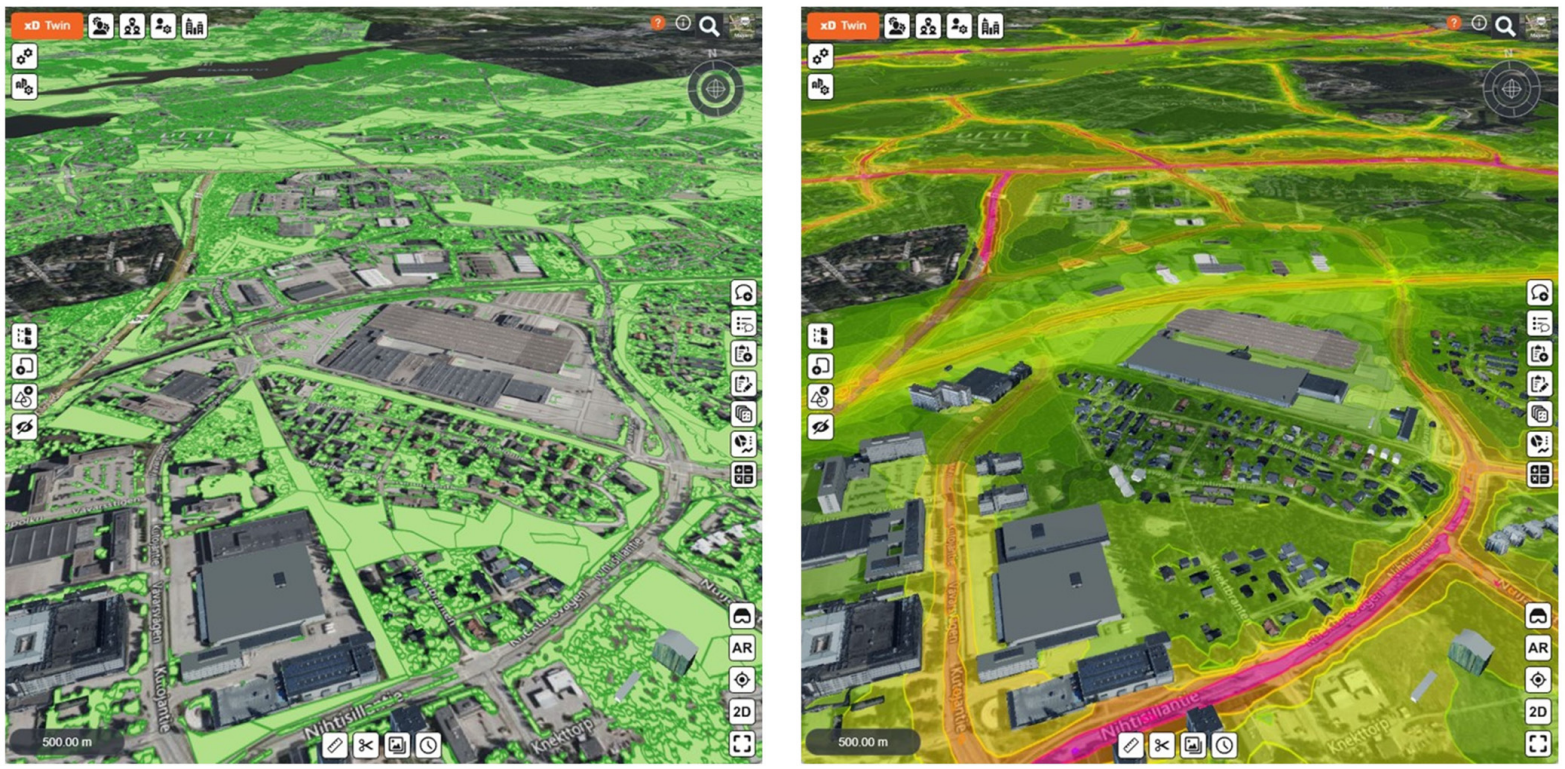
Kera digital twin in the xD Twin platform. Source: authors.

A platform called xD Twin™ by xD Visuals Ltd. was selected for the implementation of the Kera digital twin ( Figure 2). This cloud-based platform is specifically designed and developed to manage diverse urban development phases including project planning, construction, and maintenance phases in the cities´ built environment. This virtual space enables the integration of 3D city models and other data formats based on international standards such as IFC (Industry Foundation Classes) and BIM (building information models). More importantly, xD Twin also contains features for communication, including feedback collection and resident engagement, as well as a virtual space for stakeholder collaboration. Espoo´s aim to develop Kera into a sustainable and smart city district encouraged the project group to collaborate with the xD Twin developer and initiate the design work for a feature that would facilitate the integration of CO2 data from buildings and infrastructure projects into the platform. With this information, urban developers could make more informed decisions and direct, as an example, material choices towards more carbon-neutral options. Additionally, this feature could assist in calculating CO2 values for individual construction projects as well as for the entire urban district under development.

The experimentation of the xD Twin platform was kicked-off in October 2022 in collaboration with the Kera developers. The experimentation took six (6) months and was finalized in March 2023. The project group initiated evaluating the platform by exploring the datasets available from Kera and defining the goals and establishing milestones for the experimentation project. The project group was focused on investigating various aspects such as geospatial datasets, data models from buildings and infrastructure and most importantly possibility to integrate a 3D city model from Kera area into the platform.

Additionally, exploration was extended to data sets from external sources like Helsinki Region Environmental Services ( HSY, 2023) and Finnish Transport Infrastructure Agency (2023) with a focus on investigating the possibilities of importing relevant data from these data sources into the Kera digital twin. HSY data sets containing information about land covers, soil and vegetation, carbon sink assessment and real-time air quality were seen valuable and were imported to the Kera digital twin. Additionally, data sets related to noise levels from railways, streets and roads as well as data from car traffic volumes around Kera district were received and imported from Finnish Transport Infrastructure Agency into the Kera digital twin. The project group also delved into data related to pedestrians and bicycles around the existing Kera train station.

The project group also integrated building information files of the buildings to demonstrate how an urban digital twin could assist urban developers to provide a more holistic view of the specific district. Aim was to enter and explore the attribute information of the buildings in terms of material quantity, costs and CO2 emission. The buildings´ BIM information together with the infrastructure data (e.g. land covers and roads) can improve the calculation of the carbon balance of an area, but also to receive more precise information related to material quantities, costs and carbon foot/handprint of a district.

The experimentation with the xD Twin virtual platform provided valuable insights for the project group, including both Kera developers and xD Twin developer, regarding the application and deployment of digital twins and usage of data in urban development projects. Standard and neutral file formats such as city GML and IFC play a crucial role in integrating diverse datasets into virtual platforms like xD Twin and facilitating agile data sharing practises among the stakeholders using the platform. The up-to-date 3D city model, produced by the City of Espoo, proved to enhance the visual representation of a specific urban district under development, such as the Kera district. Additionally, this 3D city model forms the foundation for the creation of an urban digital twin. A visual city digital twin, supported by interoperable data formats, allow urban developers to simulate alternative and more sustainable design solutions and make informed and knowledge-based decisions that contribute to city-level sustainability efforts.

As previously mentioned, the experimentation provided promising results, but due to time constraints, the integration of citizens into this collaborative virtual environment was not realized. Additionally, experimentation of the time dimension, which would allow to observe the evolution and lifecycle of Kera area, remained limited. For xD Twin developer, the experimentation offered valuable insights on how to evolve the platform. Firstly, it improved understanding of how to automatically derive quantities and materials from IFC models for CO2 calculation purposes. Secondly, experimentation offered perspectives on how to automate CO2 calculations, as an example, by integrating CO2 calculation tool into the areal combination modelling tool. For city developers, adopting a systematic approach to deploy urban digital twin, standardized data models, and modelling practises would harmonize information flows and enhance possibilities to involve stakeholders, including citizens, in various areal development phases.

## Connecting the co-creation model and Kera digital twin

The results received from the development of the co-creation model and the urban digital twin platform experimentation in the Kera district encouraged us to organize a workshop to summarize and evaluate the outcomes of these two separate initiatives. The workshop was arranged in April 2023 for the main stakeholders (N=10) involved in the development of the co-creation model and digital twin tool experimentations. The purpose was to explore suitability of deploying a regional urban digital twin in co-creation process and to examine the potential benefits the urban digital twin may offer, including visualization, planning, and facilitating dialogue among various stakeholders. Further, the purpose was to explore the networks that emerge within the co-creation model’s processes and the virtual urban digital twin environment. Following the actor-network theory, we commence our analysis by outlining the steps of the co-creation process and depicting the main human and non-human actors found in both the co-creation process and the urban digital twin in the context of a brownfield development case ( Table 1). Thereafter, our aim is to investigate the interconnections and translations these actors form throughout the co-creation process.

**Table 1.** Combining the co-creation process with the digital platform reveals different human and non-human actors in the network.

Steps of a co-creation process	Human actors	Non-human actors	Utilization of a digital platform for the co-creation process
(1) Identification of the development need and the stakeholders	- Facilitator - City organization, city departments - Local companies and organizations, incl. service providers - Plot owners - Citizens	- Virtual platforms - 3D city model - Preliminary development ideas and plans	- Visualization of local upcoming development projects - Platform for suggesting new actions - Connecting the different stakeholders though the platform
(2) Mapping of the current situation and the development needs	- Facilitator - City organization, incl. district project manager (or similar) - Citizens - Plot owners - Data model co-ordinator	- Virtual platforms - 3D city model - Data sets	- Platform for preliminary data collection and current state key indicators - Platform for showcasing development needs from different stakeholders
(3) Setting of objectives	- Facilitator - City organization, incl. district project manager, land-use designers, city departments - Service providers - Citizens - Plot owners - Data model co-ordinator	- Virtual platform - 3D city model - Data sets - Scenario models	- Showcasing different scenarios for development - Setting and co-ordinating selected objectives
(4) Identification of solutions	- Facilitator - City organization - Service providers - Citizens - Plot owners - AEC industry - Data model co-ordinator	- Virtual platform - 3D city model - Data sets - Workshops, design sprints - Scenario models	- Identification of potentials and barriers of different solutions in different scenarios
(5) Project planning	- Facilitator - City organization - Service providers - Citizens - Plot owners - AEC industry - Data model co-ordinator	- Virtual platform - 3D city model - Data sets - Scenario models - Standardized data files - City-wide data on the effects of the project (e.g. mobility data from teleoperators)	- Showcasing the lifecycle and development of the district, visualizations - Presenting and syncing plot-level data and details for the district- level presentation - Citizen engagement tools
(6) Implementation and deployment	- Facilitator - City organization - Service providers - Citizens - Plot owners - AEC industry	- Virtual platform - 3D city model - Scenario models - Data sets - Standardized data files - IoT devices	- Dynamic visualization of the district’s lifecycle, updated to reflect changes in plans and processes - Collecting real-time data (e.g. travel behaviour) - Citizen engagement tools and interaction - Dissemination and communication

In both initiatives, the co-creation process and Kera digital twin, similar human and non-human actors were identified. In the initial stage of the co-creation process (Step 1) – *identification of the development need and the stakeholders* – we identified several main stakeholder categories, such as political decision makers and city departments under city organization, citizens, local companies and organizations, and landowners. Each of the category is made up of heterogeneous actors with diverse needs and preferences. The role of a facilitator, who brings the different stakeholders together and keeps the process on going, was considered relevant not only in the beginning of the process but in all of the different steps. In our case environment, Kera district, we also identified temporary actor networks such as local start-ups, breweries, restaurants, cultural and pop-up actors, sport and service operators, which contribute to the constantly evolving Kera area. However, the role of these actors in co-creation process was not significant due to their temporary existence in the area of Kera as the old logistics halls – which acted as the main arenas of the temporary uses of the area – were set for demolition in a rather brief time frame. Observing *the non-human actors* in Step 1, the digital tools, such as virtual platforms, were found to act as environments for gathering relevant stakeholders around the same virtual ‘table’ to visualize relevant projects, developments and trends affecting the (brownfield) urban area in question, and to set up an environment for new idea and action suggestions from the different stakeholders. These virtual platforms also serve as repositories for areal 3D city model and various data sets relevant for the development of a specific urban district.

After identifying the need(s) for district-level development and stakeholders, the Step 2 assists *mapping the current situation and the development needs.* This phase enables stakeholders to define and focus on specific development areas. To support the Step 2, we noticed two additional human actors, district manager and data model coordinator, relevant to both the co-creation process and the urban digital twin. District manager´s responsibility is to oversee and manage the process of the urban development area throughout its life cycle. Additionally, the district manager governs and co-ordinates collaboration and discussions with various city officials (land use, infrastructure, building permits, politicians etc.), engages with partners in the private and third sectors, and has mutual discussions with citizens Considering the data model co-ordinator, he/she acts as a facilitator for the use of the digital platform and is responsible for the data content, accuracy and integrity of the information models. With the facilitating support of the data model coordinator, the non-human actors (such as digital environments and data sets) can be used for a shared (preliminary) data collection and visualisation in Step 2. In the case of Kera digital twin experiment, a commercial digital platform was deployed, which enabled to import and integrate various data sets to the 3D city model to localize and spatialize the data. Through c *itizen engagement activities* in Step 2, the current role of the area and its everyday uses as well as its perceived identity can be explored to provide invaluable contexts for the development work. Participatory citizen engagement activities, such as interviews, workshops, and living labs, can be utilized to gather ideas for the area’s future development.

The gathered and generated data, and their visualization through the digital platform, can be used as a basis for Step 3, *setting of the objectives*. The urban digital twin can be used to present different development scenarios and their effects, including different timelines, which can be used as a practical point of discussion when drafting shared development targets and objectives between the relevant stakeholders for the (brownfield) urban area under development. Here also the roles of the city planners and different service providers (such as energy system operators), as well as actors from the AEC (architecture, engineering and construction) industry were recognized critical in identifying possibilities and presenting different scenarios. In the case of Kera brownfield area, private plot owners, who had signed the Kera development commitment for sustainability, formed a group of actors that hold a significant role in advancing sustainability within the Kera district.

Through shared objectives in Step 3, the *solutions can be identified* in Step 4. Methods like workshops, design sprints, round table discussions and digital tools mentioned in Table 1 assists in identifying these solutions. The key actors here are the different solution and service providers and builders. In general, similar human and non-human actors as in the previous step were seen to form the relevant stakeholders here, as the different possibilities, barriers and challenges are explored and examined with the help of the digital tools, including up-to-date data sets.

Following the Step 4, the next step in the process is the practical *project planning* (Step 5), in which the actions and their timelines are planned in detail. Here, previously mentioned digital solutions (Steps 1-4) can be used to present the lifecycle and evolution of the brownfield area, based on the set objectives and solutions. Standardized data formats, such as IFC (Industry Foundation Classes), become imperative as they enhance exchange and sharing data among different software applications used by designers in AEC (architecture, engineering, and construction service sector) industry. The digital tools can visualize the outcome of the project plan through data integration, assisting stakeholders in observing temporal and spatial aspects of the urban project. The plan can further be modified and developed through stakeholder and citizen engagement processes.

In the final step in the co-creation process, (6) *implementation and deployment*, the digital twin can be used as an updated dynamic visualization of the district’s redevelopment process and timeline, and it can be updated reflecting the updates in plans and data sets, or even updated continuously through automated real-time data gathering processes, for example, on the travel behaviour on the area. The digital twin can be used for citizen engagement activities and for dissemination and communication of the redevelopment process and its results. The gathered information can act as a basis for a new co-creation process and initiate Step 1 in a cyclical fashion.

## Discussion

Drawing on previous research on co-creation in urban development processes (e.g. Bäcklund *et al.,* 2017; Leino & Puumala, 2021) the objective of our research is firstly to enhance understanding of the methods and tools that facilitate the co-creation process and stakeholders´ participation in complex urban development projects, such as in our case brownfield area Kera and PEDs. Secondly, we seek to deepen understanding of the heterogenous networks and human and non-human actors (e.g. Rydin, 2012; Rydin & Tate, 2016) that emerge and participate in the development of sustainable urban environments. The co-creation model and virtual urban digital twin technology presented in this paper aim to foster study endeavours and enhance the knowledge of inclusivity, openness and transparency, which are identified as essential aspects in the co-creation process (e.g. Leino & Puumala, 2021; Torfing & Ansell, 2021).

Following Law (1992) the modern societies cannot exist and operate without the constant interplay of humans and technology, including digital solutions. Digital tools are already now seamlessly embedded into people`s everyday lives making it challenging to distinguish the connections in between the human and non-human actors. The ANT presented in this study facilitates our understanding of the translations and interrelationships that the actors (human & non-human) must undergo, both in co-creation process and in the virtual urban digital twin environment, to form meaningful and collaborative socio-technical networks, which expand and strengthen over the time (see Table 1). Here, we examined the human and non-human actors and interactions in between these entities. We also identified the types of connections and relationships these entities establish to form meaningful heterogeneous networks. Rydin writes that ‘within the planning consent process, the material nature of the development shapes and solidifies network inter-relationships’ ( 2012: 40–41). Here, we have also included the *virtual* as an extension for the ‘material’ as digital twins with virtual tools and platforms – which are used in a brownfield development case for co-creation process – affect and change the nature of the networks by introducing major non-human elements into the network.

In the previous section, our attempt was to provide an exemplary description of how the digital twin technologies could be used in connection with a structured co-creation process. Examining a potential brownfield development case of the Kera district through these two tools – both separately and in conjunction with one another – reveal different kinds of actor networks related to the (re)development of the area, and how they transform and change during the different phases of the brownfield area development process as different human and non-human actors are connected to the process at different stages ( Table 1). As noticed in the Kera case area, the networks, the groups of actors critical for the brownfield development process, are diverse and heterogeneous, and they do not necessarily act under the same logic in the networks in general (see Bäcklund *et al.,* 2017). The exploration of the networks reveals how the emergent networks in the brownfield development case are also strongly temporal and dynamic in nature, changing and evolving based on the process and participation of different stakeholders and non-human elements, including different data sets and digital tools. Similarly, the interactions and transactions between the different stakeholders are best understood as fluid and dynamic, taking different shapes based on the development issue at hand (see Rydin, 2012).

The experiences with the digital twin tool in Kera highlighted how the data content and needs evolves accordingly to the co-creation process. Further, during the digital twin experimentation, variations in technology acceptance among the stakeholders were identified. Some actors did not perceive the deployment of urban digital twin into existing work processes relevant, whereas the others considered urban digital twin as a robust solution for managing future urban development project with data. Additionally, concerns related to data quality, ownership, and governance as well as data privacy and security were highlighted and expressed. We also found out that a human resource, data co-ordinator, is of relevance in the digital twin assisted co-creation process as the data co-ordinator can act as an intermediary between human and non-human actors throughout the co-creation process. The integration of diverse data sets calls for standardization, which can enhance connectivity and interoperability of data across both physical and virtual realms, and which could potentially also support the stability of the network, if mutually deployed by the stakeholders.

The presented co-creation process’ steps in combination with the digital twin technologies can, ideally, be seen as a way to provide structure and form for the brownfield redevelopment process as it progresses and gathers more substance and stacking levels of interactions between the different stakeholders. The progress, though, in real life is more cyclical than linear in nature, as the idealized process can never fully be utilized in its presented form as all districts, areas, stakeholders and networks carry different narratives, histories with them. We see that the need for tools for structured co-creation processes in urban development are increasingly required in the future. Climate neutral city development and zero-carbon solutions are rarely in the hands of individual actors but are based on complex systems and actor-networks with both human and non-human elements. In the case of PEDs, for example, active collaboration is required between numerous stakeholders to achieve energy efficiency, energy use optimization and the utilization of waste energy in a specific district, which in many cases are located on multiple plots with different landowners. This increases the physical (buildings, spaces), practice-related (how solutions are used) and temporal (at what time things are used, optimized use of both during the day and the night) complexity of the urban environment from a purely technological, or technological-social, perspective. When we start to add all of the other aspects and layers of the city on top of that, things become highly complex very quickly (see Lynch, 1984).

## Conclusion

This paper has presented two practical tools for co-creation in urban development: the co-creation model and the urban digital twin. Both tools were developed and examined within the context of the brownfield area Kera, which was used as a case study environment. The study explored what these tools, when combined, could enable in a multi-stakeholder (virtually enhanced) co-creative process in terms of identified possibilities and challenges. Additionally, the paper investigated how these tools could facilitate and support the urban renewal development process of the Kera brownfield area. Furthermore, the study examines and analyses the emergent networks of the prospective co-creation process and urban digital twin through the concepts and frameworks presented in the actor-network theory. We see that both tools have the potential to reinforce urban design process and consequently assist the demonstration of alternative urban design solutions that foster sustainability across its different dimensions, including the environment, economy, social and culture. As highlighted in the text, new sustainable urban concepts – including the PED – require new tools and processes that can manage and utilize the inherent complexity of the development issues at hand involving multiple different heterogenous stakeholders.

Despite the promising advantages that the co-creation process in conjunction with urban digital twin engenders, there is a need for practical evidence from real-world urban development projects to validate their effectiveness and impacts on the stakeholder co-creation and participation. As an example, closer examination of the roles and responsibilities of the involved actors, would shed light on the management and governance of both the co-creation model and the urban digital twin piloted in Kera.

For network practitioners this study provides insights to observe changing (actor) networks within the constantly evolving virtual-physical environments, in which the human and non-human actors operate and are part of. Immersive virtual worlds are paving the way towards seamless connections between human and non-human entities both in virtual and real worlds. The utilization of these emerging and continuously developing digital technologies is dependent of the individuals and of the organization culture. The integration of new tools and methods also creates new power relations and dynamics inside the emerging networks (see Rydin & Tate, 2016). The recent sprout of artificial intelligence (AI) technologies and the evolving immersive metaverse solutions are, for example, emergent pathways in virtual-physical environment development, and will most likely have a large impact on co-creation processes in the context of urban development.

## Data Availability

No data are associated with this article.

## References

[ref-1] Adams D De Sousa C Tiesdell S : Brownfield development: a comparison of north American and British approaches. *Urban Stud.* 2010;47(1):75–104. 10.1177/0042098009346868

[ref-2] Ahvenniemi H Huovila A Pinto-Seppä I : What are the differences between sustainable and smart cities? *Cities.* 2017;60(Part A):234–245. 10.1016/j.cities.2016.09.009

[ref-3] Arnstein SR : A ladder of citizen participation. *J Am Inst Plann.* 1969;35(4):216–224. 10.1080/01944366908977225

[ref-6] Bäcklund P Häkli J Schulman H : Kansalaisosallistumisen muuttuva kenttä.In: P. Bäcklund, J. Häkli and H. Schulman (Eds.) *Kansalaiset kaupunkia kehittämässä.*Tampere: Tampere University,2017.

[ref-5] Bäcklund P Mäntysalo R : Yhdyskuntasuunnittelun teorioiden kehitys ja asukkaiden osallistumisen tarkoitus [The development of urban planning theories and the role of participation]. *Terra.* 2009;121(1):19–31. Reference Source

[ref-4] Bryson J : Brownfield gentrification: redevelopment planning and environmental justice in Spokane, Washington. *Environ Justice.* 2012;5(1):26–31. 10.1089/env.2010.0045

[ref-7] Cappai F Forgues D Glaus M : A methodological approach for evaluating brownfield redevelopment projects. *Urban Sci.* 2019;3(2):45. 10.3390/urbansci3020045

[ref-8] Casamassima L Bottecchia L Bruck A : Economic, social, and environmental aspects of Positive Energy Districts—A review. *WIREs Enery Environ.* 2022;11(6): e452. 10.1002/wene.452

[ref-9] Cheng C Albert-Seifried V Aelenei L : A systematic approach towards mapping stakeholders in different phases of PED development – extending the PED toolbox.In: J.R. Littlewood, R.J. Howlett and L.C. Jain (Eds.) *Sustainability in Energy and Buildings 2021.*London: Springer,2021. Reference Source

[ref-10] City of Espoo: The developing Kera.Website of City of Espoo,2024a. Reference Source

[ref-11] City of Espoo: The future of Kera.Website of City of Espoo,2024b. Reference Source

[ref-12] Cvetinovic M Nedovic-Budic Z Bolay JC : Decoding urban development dynamics through actor-network methodological approach. *Geoforum.* 2017;82:141–157. 10.1016/j.geoforum.2017.03.010

[ref-13] Davis A Andrew J : Co-creating urban environments to engage citizens in a low-carbon future. *Procedia Eng.* 2017;180(2017):651–657. 10.1016/j.proeng.2017.04.224

[ref-14] De Munck B : Re-assembling actor-network theory and urban history. *Urban Hist.* 2017;44(1):111–122. 10.1017/S0963926816000298

[ref-15] Derkenbaeva E Vega SH Hofstede GJ : Positive energy districts: mainstreaming energy transition in urban areas. *Renew Sustain Energy Rev.* 2022;153: 111782. 10.1016/j.rser.2021.111782

[ref-16] Elkjær LG Horst M : Rights or resources? local actor roles in ‘participation’ and ‘co-creation’ in wind energy transitions. *Energy Res Soc Sci.* 2023;97: 102966. 10.1016/j.erss.2023.102966

[ref-20] Färber A : How does ANT help us to rethink the city and its promises?In: *The Routledge Companion to Actor-Network Theory.*edited by A. Blok, I. Farías and C. Roberts. London: Routledge,2020.

[ref-18] Farías I Bender T : Urban assemblages: How actor-network theory changes urban studies.London: Routledge,2009. 10.4324/9780203870631

[ref-17] Fenwick T Edwards R : Researching education through actor-network theory.Oxford: John Wiley and Sons,2012. 10.1002/9781118275825

[ref-19] Finnish Transport Infrastructure Agency. 2023. Reference Source

[ref-21] Gaber J : Building “a ladder of citizen participation”. *J Am Plan Assoc.* 2019;85(3):188–201. 10.1080/01944363.2019.1612267

[ref-24] Hämäläinen M : Urban development with dynamic digital twins in Helsinki city. *IET Smart Cities.* 2021;3(4):201–210. 10.1049/smc2.12015

[ref-22] Healey P : The communicative work of development plans. *Environ Plann B Plann Des.* 1993;20(1):83–104. 10.1068/b200083

[ref-23] Helsinki Region Environmental Services.2023. Reference Source

[ref-25] Jacobs J : The death and life of great American Cities. 50th Anniversary Edition. New York: Modern Library,1961(2011). Reference Source

[ref-26] Latour B : On actor-network theory: a few clarifications. *Soziale Welt.* 1996;47(4):369–381. Reference Source

[ref-27] Latour B : Reassembling the social - an introduction to actor-network-theory. New York: Oxford University,2005. Reference Source

[ref-28] Law J : Notes on the theory of the actor-network: ordering, strategy, and heterogeneity. *Surv Pract.* 1992;5:379–393. 10.1007/BF01059830

[ref-29] Leino H Puumala E : What can co-creation do for the citizens? Applying co-creation for the promotion of participation in cities. *EPC: Politics and Space.* 2021;39(4):781–799. 10.1177/2399654420957337

[ref-30] Lund DH : Co-creation in urban governance: from inclusion to innovation. *Scand J Public Administration.* 2018;22(2):3–17. Reference Source

[ref-31] Lynch K : The Immature arts of city design. *Places.* 1984;1(3):10–21. Reference Source

[ref-32] Müller M : Assemblages and actor-networks: rethinking socio-material power, politics and space. *Geogr Comp.* 2015;9(1):27–41. 10.1111/gec3.12192

[ref-33] Müller M Schurr C : Assemblage thinking and actor-network theory: conjunctions, disjunctions, cross-fertilisations. *Trans Inst Br Geogr.* 2016;41(3):217–229. 10.1111/tran.12117

[ref-34] Nylén EJ Riki A Jokinen A : Kiertotalouden kestävyyslupaukset: espoon, lahden, tampereen ja turun kaupunkistrategioiden vertailu. *Yhteiskuntapolitiikka.* 2021;86(4):406–418. Reference Source

[ref-35] Puerari E De Konig JIJC Von Wirth T : Co-Creation dynamics in urban living labs. *Sustainability.* 2018;10(6):1893. 10.3390/su10061893

[ref-36] Røiseland A : Co-creating democratic legitimacy: potentials and pitfalls. *Adm Soc.* 2022;54(8):1493–1515. 10.1177/00953997211061740

[ref-37] Ruiz-Mallén I : Co-production and resilient cities to climate change. In: J. Nared and D. Bole (Eds.) *Participatory Research and Planning in Practice*. The Urban Book Series. Cham: Springer Open,2020. 10.1007/978-3-030-28014-7_1

[ref-38] Rydin Y : Using actor-network theory to understand planning practice: exploring relationships between actants in regulating low-carbon commercial development. *Plan Theory.* 2012;12(1): 23–45. 10.1177/1473095212455494

[ref-39] Rydin Y Tate L : Exploring the influence of ANT. In: Y. Rydin and L. Tate (Eds.) *Actor Networks of Planning: Exploring the Influence of Actor Network Theory*. Abingdon: Routledge,2016. 10.4324/9781315714882

[ref-40] Skaburskis A : The origin of “wicked problems”. *Plan Theory Pract.* 2008;9(2):277–280. 10.1080/14649350802041654

[ref-41] Smith RG Doel MA : Questioning the theoretical basis of current global‐city research: structures, networks and actor‐networks. *Int J Urban Reg Res.* 2011;35(1):24–39. 10.1111/j.1468-2427.2010.00940.x

[ref-42] Torfing J Ansell C : Co-creation: the new kid on the block in public governance. *Policy and Politics.* 2021;49(2):211–230. 10.1332/030557321x16115951196045

[ref-43] UN: The RIO declaration on environment and development. Report of the United Nations,1992. Reference Source

