# Supplementary material for: Co-creation Processes and Urban Digital Twins in Sustainable and Smart Urban District Development - Case Kera District in Espoo, Finland
Source: Open Res Eur. 2024 Jun 28;4:130. [Version 1] doi: 10.12688/openreseurope.17791.1 (PMC11299054; doi:10.12688/openreseurope.17791.1)
Supplement: Peer review history

# Reviewer report: Reviewer response for version 1

**DOI:** 10.21956/openreseurope.19234.r42097

**Published:** 2024-08-01

**Authors:** Rushikesh Padsala

**Affiliations:** 1 Stuttgart University of Applied Sciences - HFT Stuttgart, Stuttgart, Germany

The article "Co-creation Processes and Urban Digital Twins in Sustainable and Smart Urban District Development - Case Kera District in Espoo, Finland" by Jani Tartia and Mervi Hämäläinen explores how co-creation models and urban digital twin technologies can be integrated as tools for sustainable urban development. Using the Kera District in Espoo, Finland, as a case study, the authors demonstrate how these concepts can effectively facilitate stakeholder collaboration and enhance decision-making in urban planning. The article is well-structured and easy to read, offering insights from two key EU projects:

 SPARCS: This project explores the co-creation process for Positive Energy Districts (PEDs).

 REACT-EU: This project investigates urban digital twins as digital platforms for stakeholder engagement.

 The article presents a compelling argument for integrating collaborative processes and digital technologies, such as urban digital twins, in sustainable urban development. Although the article provides a strong foundation for future studies and practical applications, the following suggestions could help improve the existing work:

 Suggestions for Improvement

- Introduction Focus:

Current Issue: The introduction gives the impression of discussing the co-creation process and urban digital twin platforms for urban development in general, rather than focusing specifically on PEDs, as highlighted in the abstract’s reference to “a co-creation model for developing PEDs.”

 Suggestion: Refocus the introduction to specifically address co-creation and the role of urban digital twins and Actor-Network Theory (ANT) through the lens of PEDs, with sustainable urban development as the main context. This approach will align the introduction more closely with the abstract and the article's specific objectives.

- Section Titles:

Current Issue: The sections “Actor-network theory and co-creation of urban districts” and “Co-creation of urban districts through the lenses of ANT” have nearly identical titles, which can be confusing.

 Suggestion: Merge these sections for clarity and conciseness. Additionally, highlight the challenges and limitations associated with applying ANT to PEDs and sustainable urban development, as discussed in the literature. This will provide readers with a comprehensive understanding of potential obstacles and considerations for practical implementation.

- Literature Review on Urban Digital Twins:

Current Issue: The article could benefit from a more detailed examination of urban digital twins.

 Suggestion: Include a literature review on the role of urban digital twins in the development of PEDs and their use in co-creation processes. Additionally, discuss the challenges of integrating urban digital twins into these processes and explore how ANT can help overcome these obstacles. This will provide readers with a deeper understanding of both the potential and limitations of these technologies.

- Case Study: Redeveloping Urban Brownfield Areas – Case Kera:

Current Issue: The article lacks details on specific aspects of the Kera District.

 Suggestion: Provide more information on the energy infrastructure of the area and how transforming it into a PED, alongside redevelopment in other infrastructure, can help prevent issues such as gentrification.

- Kera Digital Twin Section:

Current Issue: The article mentions the goal of calculating carbon emissions and identifying carbon sinks but lacks details.

 Suggestion: Provide more details on how this calculation is conducted. Explain how CO2 values from individual construction projects were aggregated for the entire urban district under development. A system architecture diagram for the development of the urban digital twin platform would also be helpful.

- Data and Methodology:

Current Issue: There is limited information on the datasets and their handling.

 Suggestion: Include a table detailing the different datasets used, their sources, formats, units of measurement, data quality, and spatial scales. This addition would enhance the article's completeness and reproducibility.

- Role of IFC and 3D City Models:

Current Issue: The article highlights the role of Industry Foundation Classes (IFC) in enriching 3D city models but lacks specifics.

 Suggestion: Clarify whether this process was applied to all buildings in Kera. This will help readers understand the scope and applicability of the IFC in the project.

- Citizen Engagement and Platform Evaluation:

Current Issue: There are contradictory statements regarding citizen integration and platform usage.

 Suggestion: Clarify the statements about citizen engagement activities and the urban digital twin platform. Was there an evaluation of the platform's UI/UX with stakeholders? Address any inconsistencies to ensure clarity.

- Table 1 Clarifications:

Current Issue: There is insufficient detail on key indicators and scenario development.

 Suggestion: In Table 1, elaborate on the key indicators identified and how they were calculated. Provide more information on the scenario development process mentioned in Step 3.

- Discussion Section Enhancements:

Current Issue: The discussion section could be more comprehensive.

 Suggestion: Expand the discussion section to include:

 Challenges faced during the co-creation process and how they were tackled.

 How the decision-making process was established given the involvement of many heterogeneous actors with varied technology acceptance levels.

 how can ANT practically improve the decision-making process through the integration of urban digital twins in the co-creation process?

Is the case presented with sufficient detail to be useful for teaching or other practitioners?

Partly

Is the work clearly and accurately presented and does it cite the current literature?

Partly

If applicable, is the statistical analysis and its interpretation appropriate?

Not applicable

Are all the source data underlying the results available to ensure full reproducibility?

Partly

Are the conclusions drawn adequately supported by the results?

Yes

Is the background of the case’s history and progression described in sufficient detail?

Yes

Reviewer Expertise:

Urban Digital Twins, 3D City Modelling, Urban Energy Simulation, Enterprise GIS, Smart-Sustainable Cities

I confirm that I have read this submission and believe that I have an appropriate level of expertise to confirm that it is of an acceptable scientific standard, however I have significant reservations, as outlined above.

# Reviewer report: Reviewer response for version 1

**DOI:** 10.21956/openreseurope.19234.r42095

**Published:** 2024-07-26

**Authors:** Mila N Koeva

**Affiliations:** 1 PGM, University of Twente, Overijssel, The Netherlands

The document provides a comprehensive review of the Kera district’s transformation from an industrial area to a sustainable urban district. It details the progression from the early 2020s, emphasizing sustainability and circular economy targets, and highlights the involvement of various stakeholders and digital twin technologies.

 The work is clearly presented and extensively cites current literature, utilizing sources to discuss co-creation processes, actor-network theory (ANT), and urban digital twins, with references to scholars like Latour and Fenwick & Edwards, building a solid theoretical foundation.

 Statistical analysis is minimal, focusing instead on qualitative analysis to describe the co-creation model and digital twin implementation. The interpretations are appropriate for the study’s goals.

 Data sources, such as 3D city models and CO2 emissions data, are described, but the document does not provide direct access to these datasets and links to the developed platforms, limiting full reproducibility. However, thorough data integration and utilization are evident.

 Conclusions are well-supported, demonstrating the success of the co-creation process and digital twin implementation in achieving sustainability and stakeholder collaboration. The case is detailed, making it useful for the scientific community and offering valuable insights and lessons for other urban development projects.

 Overall, the document is a valuable resource for both academic and practical applications in urban development, supported by current literature and practical insights.

Is the case presented with sufficient detail to be useful for teaching or other practitioners?

Partly

Is the work clearly and accurately presented and does it cite the current literature?

Yes

If applicable, is the statistical analysis and its interpretation appropriate?

Partly

Are all the source data underlying the results available to ensure full reproducibility?

Partly

Are the conclusions drawn adequately supported by the results?

Yes

Is the background of the case’s history and progression described in sufficient detail?

Yes

Reviewer Expertise:

3D city digital twins based on geospatial technologies for improved land management and urban planning

I confirm that I have read this submission and believe that I have an appropriate level of expertise to confirm that it is of an acceptable scientific standard.

# Reviewer report: Reviewer response for version 1

**DOI:** 10.21956/openreseurope.19234.r42091

**Published:** 2024-07-26

**Authors:** Federica Gaglione

**Affiliations:** 1 University of Sannio, Benevento, Italy

The paper entitled “ *Co-creation Processes and Urban Digital Twins in Sustainable and Smart Urban District Development - Case Kera District in Espoo, Finland”* sets out the objectives and development of the research itself in a clear and well-structured way. In turn, the topic appears to be highly emerging, and this is demonstrated by the funding allocated by Europe. I found Kera digital twin very interesting as well as the combination between co-creation model and the urban digital twin can be a valid practical tool for policymakers for the development of solutions, interventions and implementation of strategies in the urban context.

 However, some suggestions are useful for improving the work:

 • The paragraph *Actor network theory and co-creation of urban districts*, although clearly described, requires the theoretical framework to present more up-to-date references given that there is only one reference dating back to 2020. In the same paragraph, I would underline the importance of which urban relationships have so far been produced between the different components of the physical, functional and morphological urban system. What results have been produced so far in the scientific framework of reference? What are the limitations present in the research to understand how this study fits into the scientific framework of reference and indirectly validate its originality?

 • In the description of the case study *Redeveloping urban brownfield areas – Case Kera* I recommend inserting further parameters such as land area and housing density of the place. Furthermore, I suggest including a brief description of the planning evolution of the area under study;

 • In the paragraph *Co-creation process for smart and sustainable urban areas – Case Kera * the administration of a questionnaire is mentioned in the co-participation process. What were the characterizing elements of the questionnaire? How were the different experts selected by the different bodies?

 • In the paragraph *Connecting the co-creation model and Kera digital twin* it would also be useful to include a summary table of the data involved (data source and unit of measurement) in the development of the Kera digital twin to have an exhaustive framework of the urban characteristics involved.

Is the case presented with sufficient detail to be useful for teaching or other practitioners?

Partly

Is the work clearly and accurately presented and does it cite the current literature?

Partly

If applicable, is the statistical analysis and its interpretation appropriate?

Not applicable

Are all the source data underlying the results available to ensure full reproducibility?

Partly

Are the conclusions drawn adequately supported by the results?

Yes

Is the background of the case’s history and progression described in sufficient detail?

Yes

Reviewer Expertise:

Urban planning; spatial analysis; GIS

I confirm that I have read this submission and believe that I have an appropriate level of expertise to confirm that it is of an acceptable scientific standard, however I have significant reservations, as outlined above.

## References

[1] : Urban Digital Twins Empowering Energy Transition: Citizen-Driven Sustainable Urban Transformation towards Positive Energy Districts. *The International Archives of the Photogrammetry, Remote Sensing and Spatial Information Sciences* .2024;XLVIII-4/W10-2024: 10.5194/isprs-archives-XLVIII-4-W10-2024-51-2024 51-56 10.5194/isprs-archives-XLVIII-4-W10-2024-51-2024

[2] : Navigating urban complexity: The transformative role of digital twins in smart city development. *Sustainable Cities and Society* .2024;111: 10.1016/j.scs.2024.105583 10.1016/j.scs.2024.105583
